# The Role of Cognitive Effort in Emotion Regulation

**DOI:** 10.1007/s42761-025-00324-x

**Published:** 2025-08-26

**Authors:** Christoph Scheffel, Anne Gärtner

**Affiliations:** 1 Differential and Personality Psychology, Faculty of Psychology, Technische Universität Dresden, Dresden, Germany; 2 Department of Education and Psychology, Freie Universität Berlin, Berlin, Germany

**Keywords:** Emotion, Emotion regulation, Cognitive effort, Regulatory flexibility, Automatic regulation

## Abstract

Emotion regulation (ER) is a dynamic, multi-stage process encompassing the identification, selection, implementation, and monitoring of ER strategies. Empirical studies on ER have increasingly focused on understanding the role of cognitive effort throughout ER processes. Cognitive effort is an essential component of various ER stages: from identifying the need to regulate emotions, through the selection and implementation of ER strategies, to the monitoring of regulatory behavior. The review highlights substantial inter-individual variability in effort expenditure across ER stages and explores the impact of cognitive costs on regulatory outcomes. To synthesize the reviewed evidence, we propose an integrative framework that outlines the potential impact of cognitive effort across the different stages of emotion regulation. Findings suggest that high effort demands can increase the likelihood of regulatory failure, perpetuating negative emotional states and impairing well-being. Conversely, automatic ER processes, while less effortful, may limit adaptability to novel emotional challenges. Understanding the interplay between cognitive effort and ER is crucial for elucidating key components of the regulatory process and their implications for individual well-being.

Psychologists have long been interested in the strategies individuals use to regulate their emotions (e.g., Schachter & Singer, 1962). Since Gross's ground-breaking articles (1998a, b), research into emotion regulation (ER) – i.e., all processes by which individuals influence which emotions they have, when they have them, and how they experience and express them (Gross, 2015b) – has increased tremendously. ER is conceptualized as a cyclic and iterative process (Gross, 2015b; Paret et al., 2011; Scherer, 2001), involving the identification of a need for regulation (*identification*), the choice of strategy (*selection*), its application (*implementation*), and the ongoing evaluation of regulatory behavior (monitoring, Gross, 2015b; Sheppes, 2020).

Early studies primarily focused on the emotional and behavioral outcomes of specific ER strategies. However, an emerging body of work has emphasized the cognitive costs associated with ER, suggesting that these strategies also require substantial mental resources (Gross, 2002; Hofstee et al., 2021; Richards, 2004; Richards & Gross, 1999, 2000; Sheppes & Meiran, 2008; Sheppes et al., 2009). ER can be understood as a form of goal-directed behavior that overlaps conceptually and neurologically with cognitive control processes (Gross, 2015a; Ochsner et al., 2012; Pruessner et al., 2020). This assumption is supported by the fact that both ER and cognitive control involve the modulation of internal states in service of current goals, often requiring the inhibition of prepotent responses and the flexible updating of mental representations. Neuroimaging studies further substantiate this overlap, showing that ER consistently recruits prefrontal regions—such as the dorsolateral and ventrolateral prefrontal cortex—that are also central to executive functions like working memory, conflict monitoring, and response inhibition (Buhle et al., 2014; Etkin et al., 2015; Morawetz et al., 2017). The role of executive functions was described in detail in the framework by Pruessner et al. (2020). Given that executive functions are resource-limited, understanding the role of cognitive effort is critical for elucidating both the effectiveness and the limitations of ER (Urry & Gross, 2010).

Despite its relevance, the role of cognitive effort in ER remains underexplored. While several theoretical models acknowledge the involvement of cognitive resources in ER (e.g., Pruessner et al., 2020; Urry & Gross, 2010), they rarely delineate how cognitive effort manifests across specific regulatory stages. Although empirical interest in this topic is growing, existing studies often fail to specify which stage of ER is being examined, making it difficult to integrate findings systematically. Moreover, the complex interplay between cognitive effort, regulatory success, and regulatory flexibility—that is, the ability to adaptively select, implement, and modify strategies—remains poorly understood. The aim of the present narrative review is to provide a systematic overview of the involvement of cognitive effort across the stages of emotion regulation (*identification*, *selection*, *implementation*, and *monitoring*) and to discuss its implications for the effectiveness and flexibility of ER processes. Particular attention will be paid to inter-individual variability, methodological challenges in measuring cognitive effort, and potential avenues for future research.

## The Construct of Cognitive Effort

Cognitive effort is not uniformly defined (Kurzban, 2016; Shepherd, 2023; Steele, 2020; Thomson & Oppenheimer, 2022; Westbrook & Braver, 2015), yet several common criteria have been identified in the literature. It is commonly understood as the extent of mental resources required to solve a cognitive task (Shenhav et al., 2017) or “the degree of engagement with demanding tasks” (Westbrook & Braver, 2015, p. 396). The investment of cognitive effort is typically experienced as aversive (David et al., 2024; Inzlicht et al., 2018; Kurzban, 2016). Importantly, cognitive effort can be conceptualized along two dimensions: the objective measurable demands of a task (objective effort), and the individual’s subjective experience or perception of these demands (subjective effort; Steele, 2020). While these two dimensions are often correlated, they can diverge substantially. For instance, individuals may perceive a task as highly effortful despite low objective demands, or vice versa (Miyake, 2001; Westbrook et al., 2019; Zhou et al., 2014). Consequently, the distinction between objective and subjective effort also has methodological implications, as cognitive effort can be assessed using both subjective and physiological measures.

Subjective measures typically involve self-report scales, capturing participants’ meta-cognitive perceptions of task difficulty, resource investment, and regulatory success (Vieira, 2016; Westbrook & Braver, 2015). However, these ratings are influenced by various internal and external factors, such as emotional experiences, motivation, or prior expectations, and may not purely reflect cognitive effort per se. Physiological measures, such as pupillometry, electromyography (e.g., corrugator activity), or heart rate variability, offer more objective indices of effortful engagement (Devine et al., 2023; Kahneman & Beatty, 1966; Piquado et al., 2010; Querino et al., 2015; Radulescu et al., 2015; Segerstrom & Nes, 2007; van der Wel & van Steenbergen, 2018). Nevertheless, physiological indices are not process-pure: they can also reflect emotional arousal, cognitive load, or executive function engagement more broadly (Cacioppo et al., 2007; Gärtner et al., 2023; Zaehringer et al., 2020). Consequently, while both subjective and physiological approaches provide valuable insights, they also present challenges in isolating the unique contribution of cognitive effort in ER.

Understanding cognitive effort is important because effort influences the choice and performance of cognitive and self-regulatory tasks (Inzlicht et al., 2018; Shenhav et al., 2017; Shepherd, 2023; Westbrook & Braver, 2013, 2015). In the context of ER, cognitive effort can directly shape the selection, implementation, and flexibility of regulatory strategies. Compared to other contextual influences on ER—such as emotional intensity or trait-level executive functioning—cognitive effort represents a dynamic factor that bridges task demands and subjective experience (Inzlicht et al., 2018; Westbrook & Braver, 2015). Unlike trait-level executive functioning, effort is a dynamic and potentially malleable construct. Moreover, growing evidence suggests that cognitive effort is not only associated with real-time regulation success (Hu et al., 2025) but may also impact long-term regulatory habits and flexibility (Braunstein et al., 2017; Pruessner et al., 2020).

## Involvement of Cognitive Effort in Emotion Regulation

**Fig. 1:**
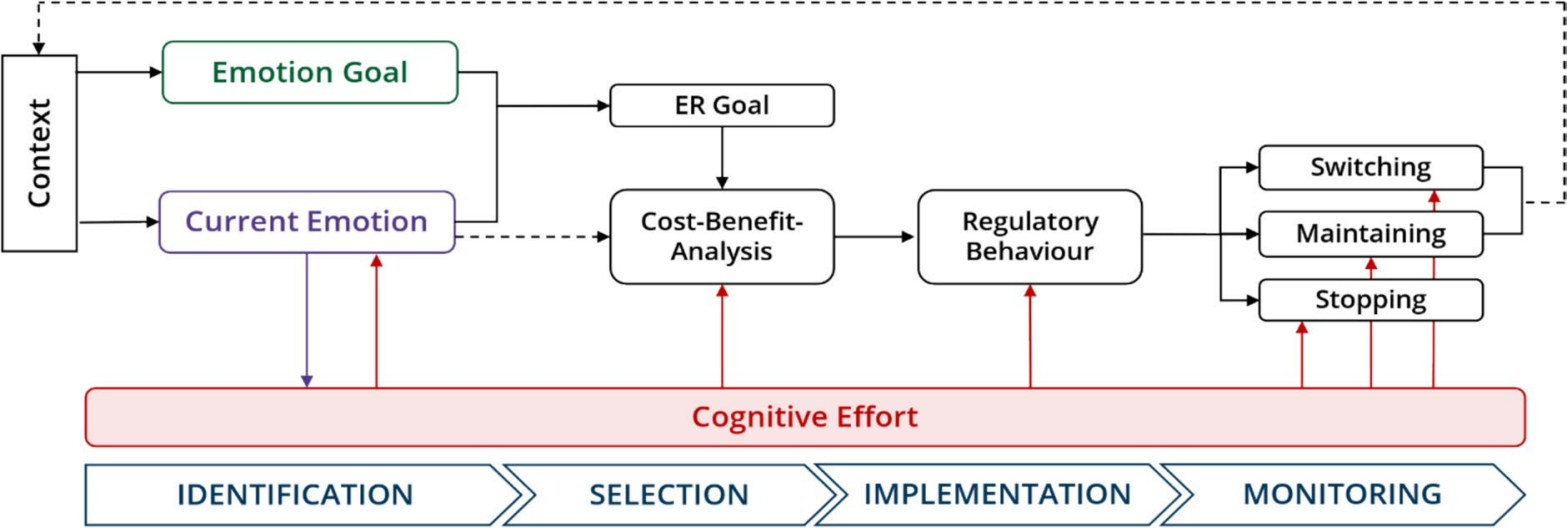
Effects of cognitive effort across stages of emotion regulation. *Note.* Overview of the potential effects of cognitive effort across the different stages of emotion regulation (i.e., identification, selection, implementation, and monitoring), as derived from the current literature. In the *identification stage*, the perception of effort is influenced by the current emotional state, while cognitive effort can in turn shape emotional experience. In the *selection stage*, cognitive effort serves as a key determinant of ER choice as it represents the costs of regulatory strategies. In the *implementation stage*, the recruitment of executive functions to apply ER strategies requires cognitive effort. Finally, in the *monitoring stage*, effort is required both to stop automatic strategies and to evaluate the necessity to maintain or switch ER strategies. The effects of effort may carry over into subsequent selection and implementation stages, as prior regulatory demands can deplete available resources

Emotion regulation is a multi-stage process involving identification, selection, implementation, and monitoring of regulatory strategies (Gross, 2015b; Sheppes, 2020). Each of these stages potentially demands cognitive effort, although the nature and degree of effort may vary. In the *identification stage*, individuals detect and conceptualize their emotional states and determine whether regulation is necessary. This introspective process relies on executive functions and can require significant cognitive effort, particularly when emotional experiences are complex or ambiguous (Lane & Smith, 2021). In the *selection stage*, individuals choose among possible ER strategies. The anticipated effort associated with different strategies serves as a key determinant of this choice (Milyavsky et al., 2019; Sheppes, 2014). According to the *Cognitive Energetics Theory* (CET; Kruglanski et al., 2012), individuals weigh the anticipated costs of regulatory effort against the expected benefits of emotional change. The *implementation stage* involves the active execution of a chosen strategy. Substantial empirical evidence demonstrates that this process requires cognitive effort, observable through subjective reports and physiological indicators such as pupillary responses (Johnstone et al., 2007; Scheffel et al., 2021). Finally, during the *monitoring stage*, individuals evaluate the effectiveness of their regulatory efforts, decide whether to maintain, adjust, or terminate regulation, and may shift strategies if necessary (Pruessner et al., 2020). These monitoring and adaptation processes also require executive control and, therefore, cognitive effort. Throughout these stages, regulatory flexibility is closely linked to the availability and management of cognitive effort. When cognitive resources are depleted or the perceived cost of regulation is too high, individuals may fail to regulate effectively or default to habitual but potentially maladaptive responses. An integrative summary of the involvement of cognitive effort across the stages of ER, including potential consequences for regulatory outcomes and flexibility, is presented in Fig. 1.

### Identification Stage

In the first stage of ER—the *identification stage*—individuals detect their current emotional state and evaluate whether regulatory action is necessary (Gross, 2015a). A crucial socio-emotional skill for successful identification is emotional awareness—the ability to recognize, differentiate, and conceptualize one’s emotional experiences (Barrett et al., 2001; Lane & Smith, 2021; Smith et al., 2018). Emotional awareness relies heavily on executive functions, such as attention regulation, working memory, and cognitive flexibility (Harlé et al., 2013). Empirical evidence directly supporting the beneficial role of effort in emotional awareness is limited. Lane and Smith (2021) assumed that greater investment of cognitive resources during the perception of emotions may enhance emotional awareness and, thereby, facilitate adaptive ER, particularly when emotions are complex, mixed, or novel. However, it could also be that higher emotional awareness is characterized by the fact that fewer resources are required to correctly detect emotional states. These possible associations between cognitive effort and emotional awareness should therefore be tested empirically.

Beyond the intrinsic cognitive demands of emotional awareness, emotional states themselves can modulate the perceived cost of cognitive effort. Individuals in negative affective states often experience cognitive tasks as more effortful (Grahek et al., 2020; Inzlicht et al., 2015). Picciotto and Fabio (2024) showed that acute stress significantly increased demand avoidance. Heightened negative emotions may thus not only increase the subjective burden of effort during emotional identification but also impair the willingness to engage in effortful regulatory processes. Indeed, in a recent ecological momentary assessment study by Lai et al. (2025), individuals reported in about 4% of the observations not wanting to engage in ER because it would require too much effort. This relatively low rate may be due to the fact that, in everyday life, people often do not experience very intense emotions (Koval et al., 2023). However, in clinical samples—where distressing emotions are more frequent and intense—the tendency to avoid regulation due to effort may be considerably more pronounced (Lai et al., 2025). This bidirectional relationship suggests that negative affect may create a vicious cycle: higher perceived effort reduces the likelihood of initiating regulation, which in turn perpetuates negative emotional experiences. Understanding the role of effort perception during emotional identification is therefore crucial for developing interventions aimed at enhancing early stages of ER.

### Selection Stage

In the *selection stage*, individuals choose an appropriate ER strategy to modulate their emotional response. Cognitive effort emerges as a key determinant of strategy choice, although this factor has only recently received empirical attention (Matthews et al., 2021). In their SOC-ER model, Urry and Gross (2010) describe how the selection of particular ER strategies is influenced by the availability of different external and internal resources. In the event of a decrease in internal resources, individuals select strategies that require other, for example external, resources.

Empirical studies have directly examined the influence of effort on ER choice behavior. For example, Sheppes et al. (2014) found that supporting the generation of reappraisal—thus reducing the generation effort—led to a higher likelihood of choosing reappraisal (Study 2). However, when emotional intensity was high, participants did not necessarily prefer less effortful strategies (Study 5), suggesting that the relationship between anticipated effort and strategy choice is context dependent. Suri et al. (2018) could show that affordances for reappraisal (i.e., how easy is it to reappraise a stimulus) are relatively stable within individuals. Although the demands interact with the intensity of the stimuli, they still significantly predict choice behavior when controlling for intensity and other contextual factors (Ortner et al., 2023; Suri et al., 2018). However, this effect appears to be specific to the choice of reappraisal, but not to the choice of distraction (Young & Suri, 2020). Moreover, there is growing evidence suggesting that this effect is not robust in interpersonal or more naturalistic contexts (Hiekkaranta et al., 2024; Matthews et al., 2022; Mitchell et al., 2025). Milyavsky et al. (2019) extended the *Cognitive Energetics Theory* (CET; Kruglanski et al., 2012) to ER choice. CET posits that goal-directed behaviors, such as choosing an ER strategy, are driven by a dynamic interplay between driving forces (e.g., goal attainment, emotional relief) and restraining forces (e.g., anticipated cognitive effort). Consequently, strategy adoption depends not only on expected effectiveness but also on cognitive cost. High effort demands can tip the balance, leading individuals to select less cognitively taxing strategies or even to forego regulation altogether. Or in other words: The adoption of a particular strategy depends on the availability of cognitive resources, with the driving force outweighing the restraining force. This complex interplay emphasises that a chosen strategy must not only be effective—anchored in the anticipation of goal achievement (the driving force)—but should also require minimal cognitive effort (the restraining force). This might lead an individual to choose a strategy that is less cognitive demanding (albeit possibly less effective) or even a complete cessation of the regulatory effort. Regarding the selection of forthcoming emotion regulation strategies, the required cognitive effort becomes intrinsically linked to the range of possible strategies that can be used in a particular context and thus influences the choice of ER.

This dynamic aligns with findings by Scheffel et al., (2021, 2023), who investigated participants’ preferences in ER strategy selection. In these studies, individuals were presented with multiple ER options and asked to justify their choice. Across all samples, the most cited reason was the anticipated lower cognitive effort. Specifically, 58% and 63% of participants in Studies 1 and 2 of Scheffel et al. (2021), respectively, and 45% in Scheffel et al. (2023) reported effort minimization as the primary motivation for their selection. Since the strategies were equally effective at the group level, the expected cognitive effort emerged as a decisive factor in strategy selection. Interestingly, this anticipated cognitive effort is represented in preparatory neural activity in event-related signals (ERPs), such as the Stimulus Preceding Negativity (SPN). Studies have shown that higher anticipated regulatory effort is associated with increased frontal brain activity before ER tasks begin (Adamczyk et al., 2024; Moser et al., 2014; Shafir et al., 2015; Thiruchselvam et al., 2011). This preparatory neural signature reflects cognitive resource mobilization in expectation of a demanding regulatory process. Anticipated effort has important consequences for regulatory flexibility. Individuals who anticipate that implementing a strategy (e.g., reappraisal) will be too effortful may switch to a less demanding alternative (e.g., distraction) even before encountering the emotional stimulus. Thus, anticipatory evaluations of effort are not passive but can actively shape strategic decisions during ER and might also serve as an antecedent to strategy switching (see section “Monitoring stage”).

In sum, these findings suggest that irrespective of theoretical effectiveness, the practical adoption of an ER strategy often depends on the cognitive effort it demands. As Tamir (2021) proposed, individuals exert regulatory effort only when the perceived benefits outweigh the costs. Thus, cognitive effort is a central determinant of ER choice. Importantly, cognitive effort also constrains regulatory flexibility: when anticipated costs are too high, individuals may avoid switching to potentially more adaptive strategies, thereby limiting their emotional adaptability.

### Implementation Stage

Empirical evidence regarding the associations between effort and ER in the *implementation stage* is much more substantial. During the implementation stage, individuals actively execute their chosen ER strategy. This phase involves the application of cognitive control processes to modify emotional experiences in line with regulatory goals. Numerous studies have demonstrated that strategy implementation demands cognitive resources and can be experienced as effortful (e.g., Gyurak et al., 2011; Kinner et al., 2017; Ortner et al., 2016; Troy et al., 2018; Webb et al., 2012). Understanding the nature and measurement of cognitive effort during this phase is critical, as it shapes both the effectiveness of regulation and the likelihood of regulatory persistence versus disengagement.

Studies have shown that individuals subjectively experience ER strategies as effortful. For example, participants often report that strategies such as reappraisal or suppression require substantial cognitive resources during active application (Moser et al., 2014; Scheffel et al., 2021, 2023; Troy et al., 2018). Importantly, it is necessary to differentiate between effort and difficulty. Effort refers to the cognitive resources invested in implementing a strategy, whereas difficulty describes the subjective sense of how challenging the regulation attempt feels. Troy et al. (2018) explicitly distinguished these two constructs in their self-report measures. They found that the ER strategies reappraisal and acceptance did not differ in perceived effort (“How hard did you try…?”), but in perceived difficulty (“How difficult was it…?”). Results implicate that these two constructs are related but separable. This distinction highlights that high subjective difficulty does not always imply high cognitive effort, and vice versa. Inter-individual differences in subjective effort ratings are substantial. For example, Scheffel et al., (2021, 2023) observed that although certain strategies on average elicited more effort than others, individuals varied greatly in their personal experiences of regulatory effort.

Physiological indicators provide complementary evidence for cognitive effort during ER implementation. Among these, pupil dilation has been consistently associated with cognitive effort during regulatory tasks (e.g., Johnstone et al., 2007; Kinner et al., 2017; Richey et al., 2015; Scheffel et al., 2021; Strauss et al., 2016; Urry et al., 2006, 2009; van Reekum et al., 2007). Larger pupil sizes typically reflect greater engagement of cognitive resources, even when overt behavior remains unchanged. Beyond pupillometry, facial electromyography (EMG) offers further insight into the exertion involved in ER. Gärtner et al. (2023) demonstrated that activity in the corrugator supercilii muscle increased even in regulate-neutral condition, suggesting a heightened engagement of cognitive control processes. This pattern of activation is interpreted as a physiological correlate of regulatory effort. This implies that EMG corrugator is not a good measure of ER success unless you control the effort, for example in balanced design with a regulate-neutral condition. Corrugator activity has been proposed to reflect the aversive component of effortful tasks (Inzlicht et al., 2018), which arise from the conflict and subjective costs imposed by cognitively demanding operations (Berger et al., 2020; Devine et al., 2023). However, it is important to note that physiological measures are not process-pure. Pupil dilation and corrugator activity can also be influenced by emotional arousal, task difficulty, or general cognitive load, making it challenging to isolate pure effort signals (see also section “The construct of cognitive effort”). Despite these limitations, converging evidence from subjective and physiological measures supports the notion that ER implementation is cognitively demanding.

Exerting cognitive effort during ER is costly, and individuals often show a tendency to avoid strategies that require substantial effort (Kool et al., 2010; Westbrook & Braver, 2013; Zerna et al., 2023). This behavioral pattern may reflect the finite nature of cognitive resources or strategic effort allocation aimed at optimizing outcomes (Baumeister et al., 1998; Kurzban et al., 2013; Muraven et al., 1998; Tamir, 2021). According to Tamir’s (2021) cybernetic control model (see also Fig. 1), regulatory effort is evaluated through a cost–benefit analysis: individuals compare their current emotional state to a desired emotional goal and assess whether the anticipated costs of effort are outweighed by the expected emotional benefit. If the perceived costs exceed benefits, individuals may reduce regulatory engagement, leading to regulatory failures. Repeated regulatory failures, in turn, can impair emotional well-being. Exhaustion of cognitive resources or a decreasing willingness to invest effort increases the risk of failed ER (Grillon et al., 2015; Hagger et al., 2010). Furthermore, heightened negative emotions can exacerbate the subjective costs of regulatory effort, reducing control engagement and perpetuating negative affect (Grahek et al., 2020; Inzlicht et al., 2015). As Tamir (2021) suggests, such dynamics can recalibrate individuals’ internal cost–benefit analyses, further decreasing future regulatory attempts and thereby negatively impacting overall well-being.

In summary, cognitive effort plays a pivotal role during the implementation of ER strategies. Subjective experiences, physiological markers, and anticipatory neural activity consistently indicate that ER implementation demands substantial cognitive resources. Importantly, the perception of high effort can influence not only the success of regulatory attempts but also individuals’ willingness to persist, adapt, or disengage from regulation, with significant consequences for emotional well-being and regulatory flexibility.

### Monitoring Stage

In the *monitoring stage* of ER, individuals continuously evaluate whether the current regulatory strategy should be maintained, adjusted, or terminated (Gross, 2015b). Monitoring involves assessing progress toward regulatory goals and comparing the current emotional state against the desired state. This stage requires ongoing engagement of cognitive control processes, particularly under conditions of high emotional intensity, and shares substantial overlap with implementation processes. However, monitoring adds the additional demand of strategic evaluation, making it an effortful and dynamic component of ER.

A key demand during the monitoring stage is maintaining the chosen ER strategy until the regulatory goal has been achieved. Especially under conditions of high emotional intensity, continuous implementation of a strategy requires sustained cognitive effort (Bonanno & Burton, 2013; Gross, 2015b; Pruessner et al., 2020 see also section “Implementation stage”). From a cognitive control perspective, maintaining regulatory behavior involves shielding the current goal from competing internal or external distractions. Shielding processes—such as inhibiting irrelevant or ruminative thoughts while implementing reappraisal (Joormann & Tanovic, 2015)—impose additional cognitive effort (Bouzidi & Gendolla, 2023; Hofmann et al., 2012; Pruessner et al., 2020; Schmeichel & Tang, 2015). Therefore, even when a strategy is already being implemented, monitoring and goal maintenance demand ongoing executive resources. High shielding effort can contribute to mental fatigue, reducing the likelihood of successfully maintaining regulatory behavior over time.

Monitoring not only involves maintaining an ongoing regulation strategy but also requires flexibility to adapt when the current strategy proves ineffective (i.e., emotion regulation flexibility). In situations where regulatory goals are not achieved, individuals may need to switch strategies to better match the emotional and contextual demands (e.g., McKone et al., 2024). For example, it was shown that impairments in switching were associated with higher levels of rumination (De Lissnyder et al., 2012). Empirical studies have shown that individuals often adaptively modify their regulatory strategies, demonstrating high regulatory flexibility. For example, switching from reappraisal to distraction when confronted with highly intense emotional stimuli has been documented as a flexible adjustment (Adamczyk et al., 2024; Birk & Bonanno, 2016; Toh & Yang, 2024). In this case, one would speak of shifting means – i.e., shifting ER strategies (Hofmann et al., 2012; Pruessner et al., 2020) to achieve a regulatory goal. Cognitive control theories indicate that task-switching operations are effortful, requiring additional executive resources to inhibit the ongoing strategy and to initiate an alternative behavior (da Silva Castanheira et al., 2021; Dreisbach & Mendl, 2024; Rubinstein et al., 2001; Yeung et al., 2006). Thus, shifting between ER strategies constitutes an effortful process that demands monitoring capacity and executive functions and flexibility (e.g., Toh & Yang, 2024). Adamczyk et al. (2024) further demonstrated that higher anticipated effort for reappraisal predicted a greater likelihood of switching to distraction versus maintaining reappraisal, suggesting that perceived cognitive costs play a role in prompting strategic shifts. However, to our knowledge, very few studies have investigated executive functions or even cognitive effort and strategy switching. Most of the studies relate to the selection process.

Lastly, monitoring involves determining when to terminate ER efforts. Once the regulatory goal has been achieved—meaning that the current emotional state aligns with the desired emotional state—continuing regulation is no longer necessary (Gross, 2015b). Termination of regulatory behavior may also occur when the perceived cognitive costs outweigh the expected emotional benefits (Tamir, 2021). In such cases, disengaging from regulation can be a strategic decision based on a cost–benefit evaluation. Importantly, stopping an ongoing regulatory process is not effortless. Neural and behavioral studies suggest that terminating habitual or ongoing actions requires inhibitory control and active cognitive engagement (e.g., Aziz-Safaie et al., 2024; Hervault & Wessel, 2024). Thus, disengaging from ER, particularly when strategies have become habitual or automatic, imposes additional cognitive effort.

In sum, the monitoring stage of ER is a dynamic and cognitively demanding process. It requires continuous assessment of regulatory success, sustained maintenance of regulatory goals, flexible switching between strategies when needed, and effortful termination of regulation. Throughout these processes, cognitive effort not only influences regulatory success but also determines the flexibility and adaptability of emotional responses.

## Effortless Emotion Regulation

While cognitive effort plays a critical role in ER, not all regulatory processes are consciously effortful. Increasing attention has been paid to effortless or automatic forms of ER, where strategies are applied with minimal conscious resource investment (Etkin et al., 2015; Gyurak et al., 2011; Rodriguez & Kross, 2023; Troy et al., 2018). Automatic ER processes can emerge through extensive practice, habitualization, or situational automatization. Over time, frequently used strategies like reappraisal or acceptance may become less effortful, as their cognitive demands decrease through repeated activation and consolidation (Ford & Troy, 2019; Mauss et al., 2007; Troy et al., 2018). Neuroimaging studies support this notion, showing reduced activation in cognitive control regions when trained individuals engage in practiced regulatory strategies (Paret et al., 2011; Troy et al., 2018). Importantly, effortless regulation is not inherently superior. Although lower cognitive costs can enhance sustainability of regulation over time, automatic processes may be less flexible and less sensitive to contextual demands (Dore et al., 2016; Ford & Troy, 2019). Thus, while automatization reduces immediate effort, it may also constrain the ability to adapt strategies dynamically. Understanding the balance between effortful and effortless ER is crucial for advancing emotion regulation models. Particularly, the transition from deliberate, effortful regulation to more automatic forms offers insights into how training and experience shape the effectiveness and flexibility of ER over time.

## Future Directions

### Differentiating Cognitive Effort Across Emotion Regulation Phases

Future research should investigate the dynamic involvement of cognitive effort across different stages of ER. Particularly, the identification stage remains relatively underexplored (Lane & Smith, 2021; Smith et al., 2018). For example, findings indicate that an increased anticipatory effort of reappraisal leads to an alternative selection of the reappraisal process in the selection stage (Adamczyk et al., 2024). However, Sheppes et al., (2014, Study 2) found increased selection of reappraisal (compared to distraction), when the generation process of reappraisal was facilitated and thereby the effort was reduced. In addition, Gutentag and Tamir (2022) found that rendering an ER goal more desirable motivates individuals to invest more effort and can boost ER success. This might inform interventions to reduce cognitive load during particularly effortful strategies or stages of ER. Studies are needed to clarify how executive functions contribute to emotional awareness, and how perceived effort during emotion perception influences subsequent regulation choices. Moreover, anticipated and actual cognitive effort should be systematically integrated into models of ER choice. While the *Cognitive Energetics Theory* (CET; Milyavsky et al., 2019; Tamir, 2021) has been applied to ER, direct experimental manipulation of anticipated effort is still rare. Research should explore how varying anticipated costs influence strategy selection and flexibility, particularly under different emotional intensities (Scheffel et al., 2021, 2023; Sheppes et al., 2014). Methodological improvements are also crucial: Current subjective and physiological measures (e.g., pupil dilation, corrugator activity) often overlap with constructs such as emotional arousal and perceived difficulty (Gärtner et al., 2023; Troy et al., 2018). Multimethod approaches—combining self-report, behavioral, physiological, and neural data (e.g., EEG SPN components)—could help disentangle effort-specific effects across ER phases.

### Impact of Individual Differences on Cognitive Effort

The studies by Scheffel et al., (2021, 2023) revealed substantial inter-individual differences in the subjective experience of effort during the implementation of ER strategies. Future research might focus on identifying personality traits, cognitive styles, or genetic factors that influence the relationship between cognitive effort and successful ER. For example, several traits have been linked to the investment of effort in goal-directed behavior (Kahneman, 1973; Strobel et al., 2020): The trait Need for Cognition (Cacioppo & Petty, 1982) has been associated with a preference for more effortful tasks (Westbrook & Braver, 2013; Zerna et al., 2023). Additionally, individuals with higher self-control tend to exhibit more effective ER (Paschke et al., 2016) and assign higher subjective value to ER strategies (Scheffel et al., 2023). Subjective values are indeed related to self-reported effort; however, the exact relationship between self-control and regulatory effort during ER still requires further empirical investigation.

### Clinical Implications

Emotion regulation difficulties are a core feature across numerous psychological disorders and have been identified as a transdiagnostic risk factor that emerges early in life (Saccaro et al., 2024). Regulatory failures are especially likely when cognitive effort required for regulation exceeds available cognitive resources (Grillon et al., 2015; Tamir, 2021). This suggests that cognitive effort is not merely a barrier but a potential treatment target. Established treatments such as Cognitive Behavioral Therapy (CBT) and Dialectical Behavior Therapy (DBT), but also Acceptance and Commitment Therapy (ACT) have shown robust effects in reducing emotion dysregulation across adult clinical populations, for example with depression, PTSD, or high chronic stress (Saccaro et al., 2024). Yet these treatments only show small to medium effect sizes at best (Saccaro et al., 2024). Interestingly, these populations are characterized by impaired cognitive functioning (e.g., Clausen et al., 2017; James et al., 2023; Kriesche et al., 2023; Schuitevoerder et al., 2013; Wolff et al., 2021), which suggests that explicitly addressing both perceived and actual effort in ER could be a promising approach to enhance engagement and improve long-term therapy outcomes. For example, cognitive reappraisal, though initially effortful, can become more automatic with repeated practice (Braunstein et al., 2017). Individuals with depression may be a particularly suitable target population, as current research indicates that increased regulatory effort in these individuals is not associated with regulatory success (Hu et al., 2025). Interventions could therefore focus on scaffolding the development of effective strategies while gradually increasing their cognitive demands, in order to foster resilience without overwhelming individuals’ capacity. Future research should identify which effort-related mechanisms mediate treatment outcomes, and whether modifying effort perception or strategy training leads to sustained improvements. There is also a need to test these effort-focused enhancements in underrepresented groups, including children and adolescents, where existing evidence remains limited (Saccaro et al., 2024).

## Conclusion

This narrative review highlights the central role of cognitive effort across the stages of emotion regulation—*identification*, *selection*, *implementation*, and *monitoring*. Cognitive effort emerges as a key determinant of regulatory success, flexibility, and efficiency, mediating the relationship between cognitive control, regulatory costs, and emotional well-being. Importantly, effort demands vary across stages and are shaped by individual differences, contextual factors, and the specific strategies employed. Effortful regulation, while often effective, is constrained by limited cognitive resources, potentially leading to regulatory failure and negative emotional outcomes. Conversely, automatic regulation may reduce cognitive demands but can limit flexibility in complex contexts. Cognitive effort also plays a crucial role in regulatory flexibility, influencing individuals’ ability to adaptively select and switch strategies. However, several ER stages remain underexplored regarding effort. In many cases, the influence of effort is inferred from broader measures of executive function rather than assessed directly; in others—such as strategy switching—empirical evidence is lacking altogether. This review helps identify these gaps and offers a framework for future empirical work. Future research should address the methodological challenges of measuring effort, ideally using multimethod approaches that distinguish effort from related constructs like arousal or perceived difficulty. In parallel, interventions aimed at optimizing perceived and actual effort—particularly in clinical and high-stress populations—may enhance emotional resilience and psychological well-being. By integrating cognitive effort more explicitly into emotion regulation models, this review advances theoretical understanding and lays the groundwork for novel empirical and clinical applications.

## Additional Information

### Availability of data and material

No datasets were generated or analysed during the current study.
